# Supplementary material for: CTCF Expression is Essential for Somatic Cell Viability and Protection Against Cancer
Source: Int J Mol Sci. 2018 Nov 30;19(12):3832. doi: 10.3390/ijms19123832 (PMC6321389; doi:10.3390/ijms19123832)
Supplement: Supplementary file 1 [file ijms-19-03832-s001.pdf]

Supplementary Figure Legends

Supplementary Figure 1 CRISPR/Cas9 targeting strategy in K562 and *Ctcf*$^{+/pgkneo}$ MEFs. A) Schematic of lentiviral vectors used for CRISPR/Cas9 disruption of *CTCF*: LTR=long terminal repeat; cppt=central polypurine tract; RRE=rev response element; CAGGS=CMV early enhancer/chicken β-actin promoter; Bsd=blasticidin resistance gene; 2A=picornaviral 2A peptide sequence; UbC=ubiquitin C promoter; 3FLAG=3xFLAG tag. B) Genomic location of sgRNAs used to target human *CTCF* exon 3; primers used to amplify the targeted region are shown with half-arrowheads. C) Location of sgRNAs used to target mouse *Ctcf* exon 3. Primers used to amplify the targeted region are shown with half-arrowheads. Flow cytometry plots of K562 cells D) and *Ctcf* $^{+/pgkneo}$ MEFs E) showing efficient transduction with Cas9 (eGFP) and sgRNA (mCherry) vectors.

Supplementary Figure 2 Ctcf expression in hemizygous *Ctcf* MEF clones after CRISPR/Cas9 editing. FACS-enriched *Ctcf*$^{+/pgkneo}$ MEFs after *Ctcf* inactivation using CRISPR/Cas9 genome editing were analysed for Ctcf expression by Western blot. Representative blots showing Ctcf protein expression in clones containing *Ctcf* sgRNA#1, sgRNA#2, sgRNA#3 or the control sgRNA targeting *Rosa26*. WT and *Ctcf*$^{+/pgkneo}$ (het) MEFs were included as controls. Arrowheads indicate clones that have lower molecular weight Ctcf species resulting from microdeletions or alternative downstream start codon usage as consequence of upstream frameshift mutations.

Supplementary Figure 3 Differentially expressed genes in *CTCF*-altered endometrial cancer. Analysis of mRNA expression in genes differentially expressed between *CTCF*-altered and *CTCF* normal (diploid) endometrial cancer. Plots show gene expression of selected genes from a 642 differentially expressed gene signature (q<0.05); * indicates those genes that were not in the signature. A) *CTCF*; B) *TP53,* red filled circles indicate samples with *TP53* mutations; C) *TP53* with *TP53* mutant samples removed; D) *CTCFL*; E) *H19*; F) *ZFHX3*; tumour suppressor genes: G)

CDKN2A; H) *PIK3CA*; I) *CDH6*; J) *IGF2BP2*; as well as estrogen-responsive genes K) *KIAA1324*; L) *MLPH*; M) *MSX2*; N) *SPDEF*; O) *TFF3*; P) *PIGR*. Data represents the mean±S.D. with statistical analysis performed using the Student's t-test for *CTCF* WT EC (n=187) and *CTCF*-altered EC (n=45).

Bailey_Supplementary_Figure_1

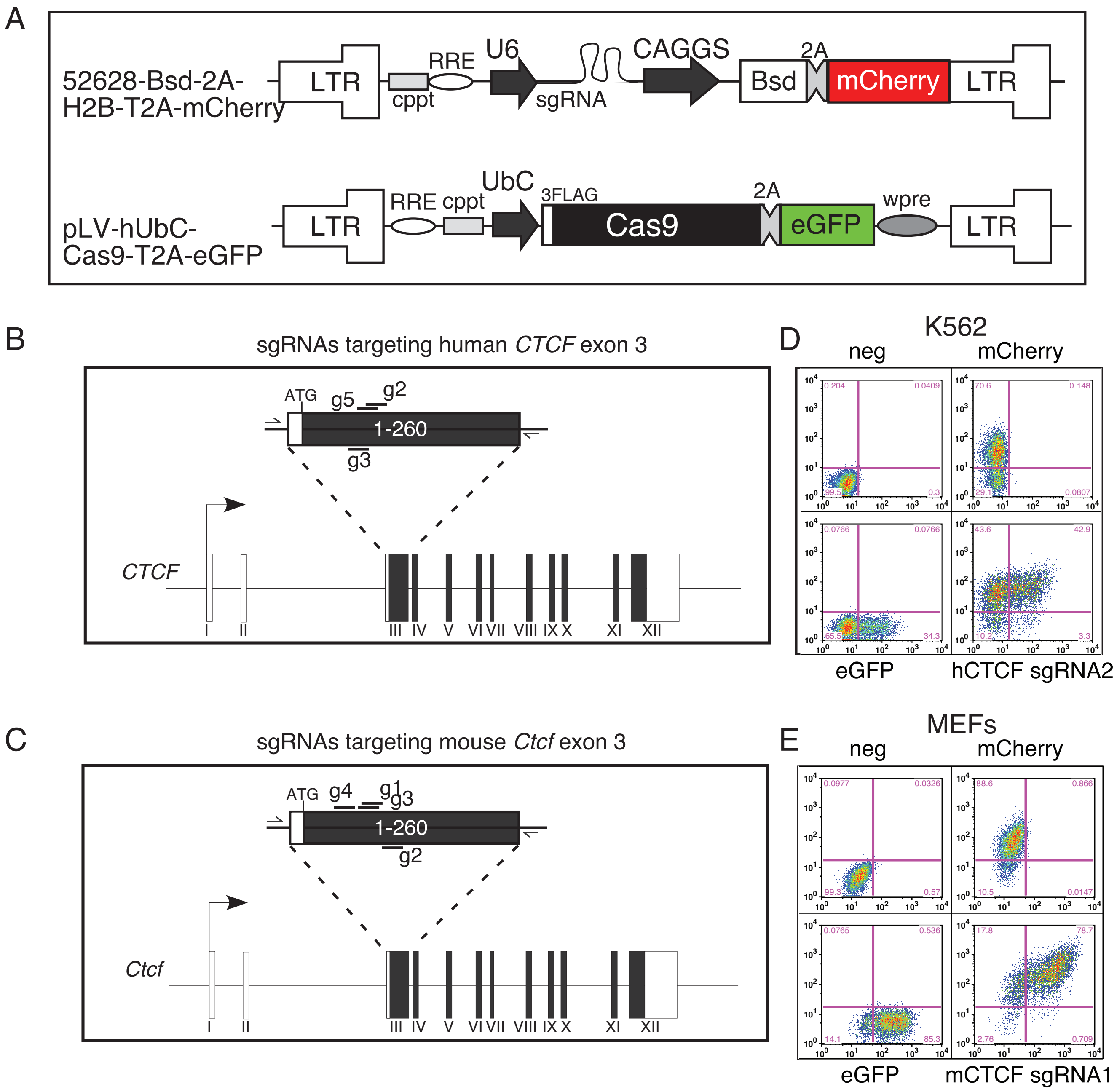

Supplementary Figure_2

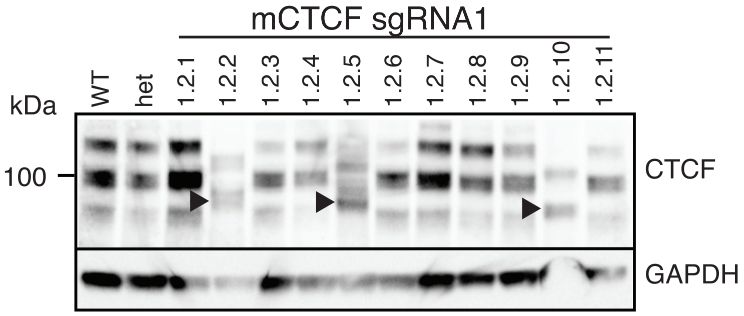

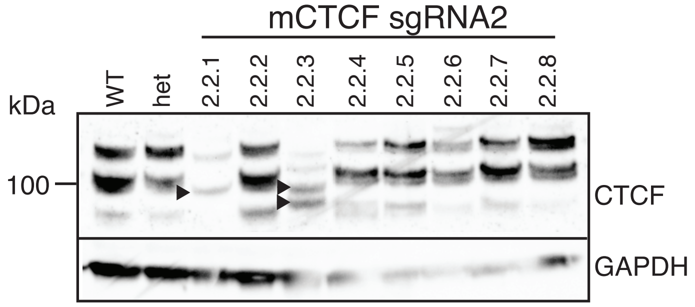

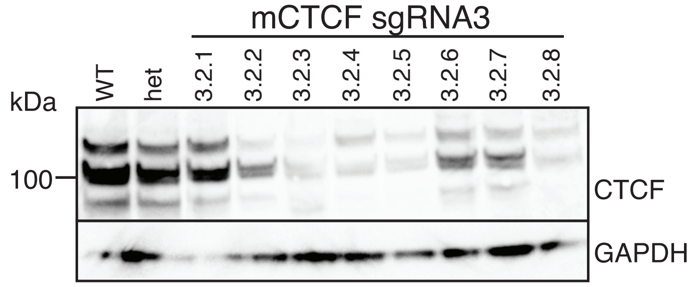

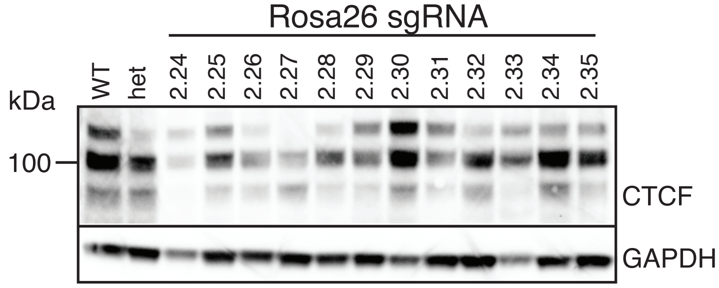

Supplementary Figure 3

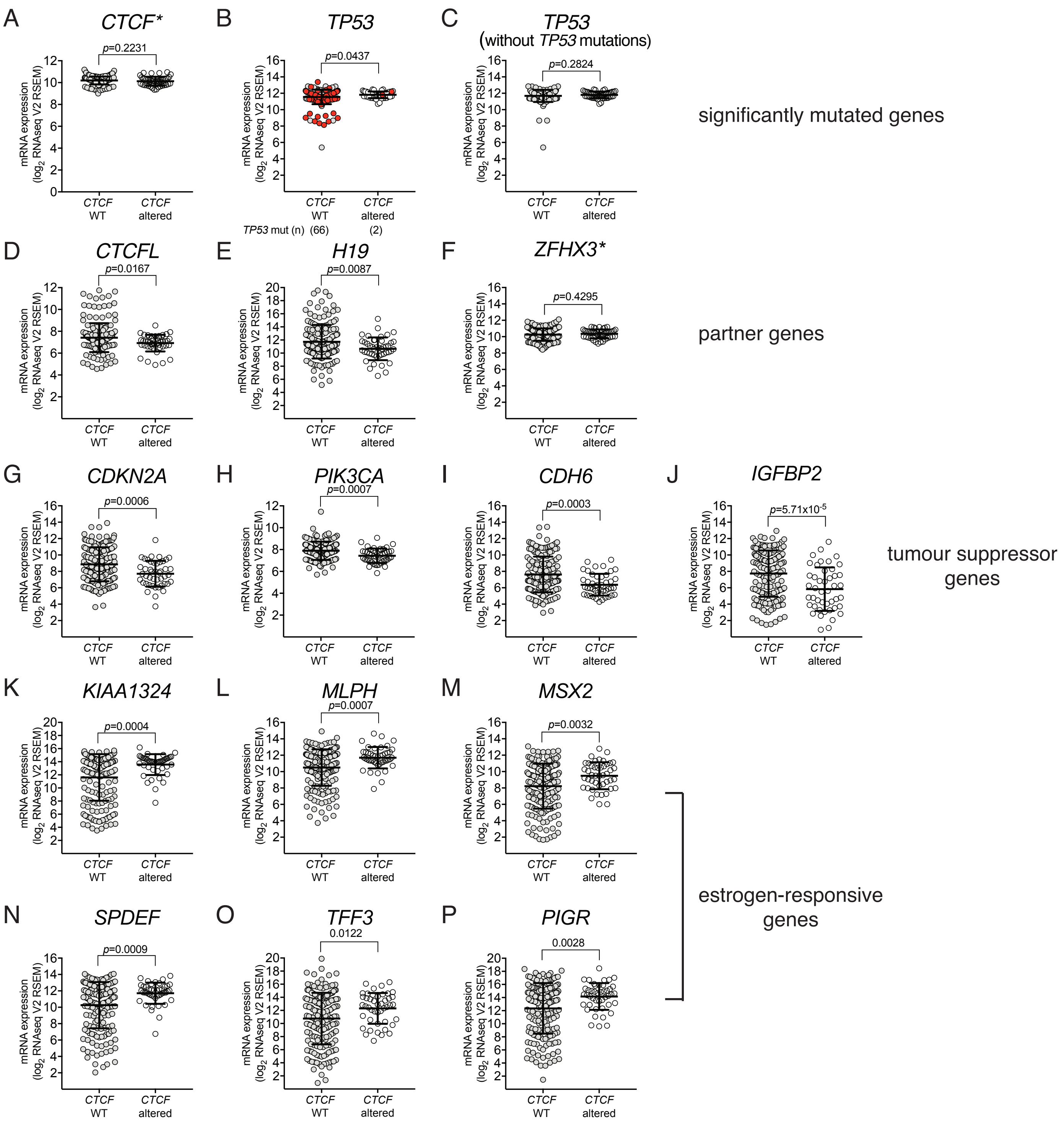

Supplementary Table 1: Oligonucleotides used in this study.

Application	Name	Sequence	Purpose
CRISPR/Cas 9 sgRNA	hCTCF-gRNA2-s	accgAGGAACAGCCCATAAACATAGG	sgRNA oligo that targets human *CTCF* coding exon 3; clone into *BspMI* sites
	hCTCF-gRNA2-as	aaacCCTATGTTTATGGGCTGTTCCT	
CRISPR/Cas 9 sgRNA	hCTCF-gRNA3-s	accgAACCTGTAAAGTTATAATCTGG G	sgRNA oligo that targets human *CTCF* coding exon 3; clone into *BspMI* sites
	hCTCF-gRNA3-as	aaacCCCAGATTATAACTTTACAGGTT	
CRISPR/Cas 9 sgRNA	hCTCF-gRNA5-s	accgAACTTTACAGGTTGTAAATATG G	sgRNA that targets human *CTCF* coding exon 3; clone into *BspMI* sites
	hCTCF-gRNA5-as	aaacCCATATTTACAACCTGTAAAGTT	
PCR	hCTCF-T7E1-5'	AATAAAGGCAGGGGAAATGG	655 bp in human *CTCF*; confirmation of gene editing using T7 endonuclease I
	hCTCF-T7E1-3'	ACGCAGTTTGCTCTTTTTGG	
CRISPR/Cas 9 sgRNA	AAVS1-gRNA-s	accgACCCCACAGTGGGGCCACTA	sgRNA that targets AAV integration site AAVS1; clone into *BspMI* sites
	AAVS1-gRNA-s	aaacTAGTGGCCCCACTGTGGGGT	
PCR	AAVS1-T7E1-5'	AGGTTCTGGGAGAGGGTAGC	668 bp in human AAVS1; confirmation of gene editing using T7 endonuclease I
	AAVS1-T7E1-3'	CTGGACAACCCCAAAGTACC	
CRISPR/Cas 9 sgRNA	mCTCF-gRNA1-s	accgAACCTGCAAGGTTATGATCT	sgRNA that targets mouse *CTCF* coding exon 3; clone into *BspMI* sites
	mCTCF-gRNA1-as	aaacAGATCATAACCTTGCAGGTT	
CRISPR/Cas 9 sgRNA	mCTCF-gRNA2-s	accgGAGGAACAGCCCATTAACAT	sgRNA that targets mouse *CTCF* coding exon 3; clone into *BspMI* sites
	mCTCF-gRNA2-as	aaacATGTTAATGGGCTGTTCCTC	
CRISPR/Cas 9 sgRNA	mCTCF-gRNA3-s	accgCAACCTGCAAGGTTATGATC	sgRNA that targets mouse *CTCF* coding exon 3; clone into *BspMI* sites
	mCTCF-gRNA3-as	aaacGATCATAACCTTGCAGGTTG	
CRISPR/Cas 9 sgRNA	mCTCF-gRNA4-s	accgTCCACTGCAGCCTCTGCTTC	sgRNA that targets mouse *CTCF* coding exon 3; clone into *BspMI* sites
	mCTCF-gRNA4-as	aaacGAAGCAGAGGCTGCAGTGGA	
PCR	mCTCF-T7E1-5'	TGCCTGTGGGTTCTAAGTGC	641 bp in mouse *CTCF* coding exon 3; confirmation of gene editing using T7 endonuclease I
	mCTCF-T7E1-3'	TGAAATCCTTCAGGCAAAGG	
CRISPR/Cas 9 sgRNA	Rosa26-gRNA-s	accgACTCCAGTCTTTCTAGAAGA	sgRNA that targets mouse *Rosa26*. Clone into *BspMI* sites
	Rosa26-gRNA-as	aaacTCTTCTAGAAAGACTGGAGT	
PCR	Rosa26-T7E1-5'	CGTGCAAGTTGAGTCCATCCGCC	749 bp in mouse *Rosa26*; confirmation of gene editing using T7 endonuclease I
	Rosa26-T7E1-3'	ACTCCGAGGCGGATCACAAGCA	
CTCF$^{+/-}$ mouse genotyping	CTCFwt-5'	TGGGCTCTATGGCTTCTGAG	Detects wildtype allele 519 bp and knockout allele 348 bp in mouse *CTCF*
	CTCFwt-3'	CATGCCATCCTACTGGTGTG	
	CTCF(0)-3'	CTCACGCCTGAGATGATCC	
